# Development of an Artificial Intelligence Model for the Classification of Gastric Carcinoma Stages Using Pathology Slides

**DOI:** 10.7759/cureus.56740

**Published:** 2024-03-22

**Authors:** Shreya Reddy, Avneet Shaheed, Yui Seo, Rakesh Patel

**Affiliations:** 1 Biomedical Sciences, Creighton University, Omaha, USA; 2 Pathology, University of Illinois at Chicago, Chicago, USA; 3 Medicine, California Northstate University College of Medicine, Elk Grove, USA; 4 Internal Medicine, East Tennessee State University Quillen College of Medicine, Johnson City, USA

**Keywords:** treatment of gastric cancer, artificial intelligence (ai), gastric pathology, tumor staging, gastric tumor

## Abstract

This study showcases a novel AI-driven approach to accurately differentiate between stage one and stage two gastric carcinoma based on pathology slide analysis. Gastric carcinoma, a significant contributor to cancer-related mortality globally, necessitates precise staging for optimal treatment planning and patient management. Leveraging a comprehensive dataset of 3540 high-resolution pathology images sourced from Kaggle.com, comprising an equal distribution of stage one and stage two tumors, the developed AI model demonstrates remarkable performance in tumor staging. Through the application of state-of-the-art deep learning techniques on Google's Collaboration platform, the model achieves outstanding accuracy and precision rates of 100%, accompanied by notable sensitivity (97.09%), specificity (100%), and F1-score (98.31%). Additionally, the model exhibits an impressive area under the receiver operating characteristic curve (AUC) of 0.999, indicating superior discriminatory power and robustness. By providing clinicians with an efficient and reliable tool for gastric carcinoma staging, this AI-driven approach has the potential to significantly enhance diagnostic accuracy, inform treatment decisions, and ultimately improve patient outcomes in the management of gastric carcinoma. This research contributes to the ongoing advancement of cancer diagnosis and underscores the transformative potential of artificial intelligence in clinical practice.

## Introduction

Gastric carcinoma, commonly known as stomach cancer, arises from the epithelial cells lining the stomach mucosa [1]. It is a multifactorial disease with a complex etiology and is influenced by a combination of genetic, environmental, and lifestyle factors [1]. The formation of gastric carcinoma typically follows a stepwise progression from normal gastric mucosa to precancerous lesions, and eventually to invasive carcinoma [2].

The development of gastric carcinoma is often preceded by chronic inflammation of the gastric mucosa, a condition known as chronic gastritis [2]. Chronic gastritis can be triggered by various factors, including infection with *Helicobacter pylori *bacteria, long-term use of nonsteroidal anti-inflammatory drugs (NSAIDs), excessive alcohol consumption, and tobacco use [2,3]. Prolonged exposure to these risk factors can lead to the accumulation of genetic mutations and genomic instability within the gastric epithelial cells, setting the stage for carcinogenesis [4]. In some cases, chronic gastritis may progress to the development of precancerous lesions, such as gastric atrophy, intestinal metaplasia, and dysplasia [5]. These lesions represent intermediate stages in the cascade of gastric carcinogenesis and are associated with an increased risk of developing invasive carcinoma [6]. Notably, the presence of *H. pylori *infection is strongly correlated with the development of precancerous lesions, highlighting the role of this bacterium as a major risk factor for gastric carcinoma [4]. As the disease progresses, invasive carcinoma may arise from the transformed epithelial cells, leading to the formation of malignant tumors within the stomach wall [2].

Gastric carcinoma can manifest in various histological subtypes, including intestinal-type adenocarcinoma, diffuse-type adenocarcinoma, and other rare histological variants [7]. The clinical presentation of gastric carcinoma often includes nonspecific symptoms such as abdominal pain, dyspepsia, weight loss, and gastrointestinal bleeding, which can contribute to delays in diagnosis and treatment [8]. Given the diverse etiology and histopathological characteristics of gastric carcinoma, accurate diagnosis and staging are essential for guiding appropriate therapeutic interventions and improving patient outcomes. Histopathological examination of tissue specimens, particularly through analysis of pathology slides, remains the gold standard for diagnosing and staging gastric carcinoma [9]. However, this process is labor-intensive, time-consuming, and subject to interobserver variability, underscoring the need for objective and efficient methods of tumor classification. This study aims to address this need by developing an artificial intelligence (AI) model capable of differentiating between stage one and stage two gastric carcinoma using pathology slides, thereby enhancing diagnostic accuracy and efficiency.

Distinguishing between stage one and stage two gastric carcinoma is crucial for treatment planning and prognostic assessment, as these stages exhibit distinct characteristics and clinical implications [10]. Stage one tumors typically remain localized within the mucosal or submucosal layers of the stomach wall, presenting with well-defined glandular structures and minimal cellular atypia, often resulting in favorable treatment outcomes and prognosis [11,12]. Conversely, stage two tumors demonstrate deeper invasion into the gastric wall, extending into the muscularis propria or even penetrating through the serosa, accompanied by increased cellular proliferation, nuclear atypia, and architectural disorganization [13]. This advanced stage is associated with a higher risk of lymph node involvement and metastasis, highlighting the importance of accurate staging for guiding therapeutic decisions and predicting patient outcomes [14]. Histological evaluation plays a pivotal role in distinguishing between these stages, providing valuable insights into tumor behavior and aggressiveness, thereby informing individualized treatment strategies tailored to the specific needs of each patient [12]. Understanding these distinctions is essential for optimizing patient care and improving clinical outcomes in the management of gastric carcinoma.

Artificial intelligence (AI) has emerged as a valuable tool in the diagnosis, staging, and management of gastric carcinoma, offering several significant contributions to clinical practice [15]. Firstly, AI-based algorithms have demonstrated efficacy in the detection of gastric lesions and early-stage carcinomas on endoscopic images, aiding in the identification of suspicious areas for biopsy and histological confirmation [16]. Additionally, AI-driven models have shown promise in predicting patient prognosis and treatment outcomes by analyzing histopathological features and molecular biomarkers associated with gastric carcinoma [17]. Furthermore, AI-powered image analysis techniques have facilitated the accurate classification and staging of gastric tumors, providing valuable insights into tumor characteristics and guiding personalized treatment strategies [18]. Moreover, AI algorithms have the potential to streamline diagnostic workflows, reduce interobserver variability, and improve efficiency in the diagnosis and management of gastric carcinoma [18]. Overall, the integration of AI technologies into clinical practice holds great promise for enhancing the accuracy, efficiency, and effectiveness of gastric carcinoma care [18].

Clinically, the distinction between stage one and stage two gastric carcinoma has implications for treatment selection and patient prognosis. Stage one tumors may be amenable to curative surgical resection alone, with favorable long-term survival rates [19]. In contrast, stage two tumors may require more extensive surgical interventions, such as subtotal or total gastrectomy with lymphadenectomy, and may benefit from adjuvant therapies such as chemotherapy or radiation therapy to reduce the risk of recurrence [19]. Additionally, the prognosis for patients with stage two gastric carcinoma may be influenced by factors such as tumor size, depth of invasion, histological grade, presence of lymphovascular invasion, and degree of lymph node involvement [19]. Comprehensive staging and accurate classification of gastric carcinoma are therefore essential for guiding treatment decisions, predicting patient outcomes, and optimizing overall survival. The aim of this study is to create and assess an artificial intelligence (AI) model proficient in precisely discerning between stage one and stage two gastric carcinoma using pathology slides. By leveraging artificial intelligence (AI) technology to differentiate between stage one and stage two gastric carcinoma based on pathology slides, this study aims to improve the accuracy and efficiency of tumor staging, ultimately enhancing patient care and clinical management.

## Materials and methods

Pathology slides depicting gastric carcinoma samples from patients with stage one and stage two tumors were collected for this study. The dataset utilized in this research was sourced from Kaggle.com, a widely recognized platform for accessing and sharing datasets for research and educational purposes. The dataset selection for this study adhered to stringent criteria to ensure its appropriateness and reliability. Specifically, datasets from Kaggle.com, a reputable platform for research and educational datasets, were chosen due to their high-quality and publicly accessible nature. The dataset comprised a total of 3540 high-resolution images, consisting of pathology slides of stage one gastric carcinoma and pathology slides of stage two gastric carcinoma. The composition of the dataset is summarized in Table 1.

**Table 1:** Dataset Composition

Subset	Amount of Images
Stage One Gastric Carcinoma	1770 (50%)
Stage Two Gastric Carcinoma	1770 (50%)
Total	3540

Regarding resolution, all images were standardized to ensure consistency across the dataset, thereby minimizing potential confounding factors during model training and evaluation. Imbalances in the dataset, such as variations in the number of images between different stages of gastric carcinoma, were addressed through careful handling. Specifically, a stratified random sampling approach was employed during the dataset-splitting process to ensure that each subset (training, validation, and testing) contained a proportional representation of images from both stage one and stage two gastric carcinoma. This method helped prevent biases in model training and evaluation, enhancing the robustness and generalizability of the AI model. The dataset's comprehensive nature ensures a balanced representation of different tumor characteristics and histological features, enabling the AI model to learn and generalize effectively. Each image in the dataset undergoes meticulous preprocessing to enhance quality and standardization, facilitating accurate and reliable tumor classification.

To ensure robustness and generalizability, the dataset was randomly split into three distinct subsets: training, validation, and testing. The split of the dataset is outlined in Table 2. The training set was used to optimize the AI model's parameters and learn the underlying patterns associated with differentiating between stage one and stage two gastric carcinoma on pathology slides. The validation set served as an independent dataset for evaluating the model's performance during the training process and tuning hyperparameters to prevent overfitting. The validation set allowed for iterative refinement of the model's architecture and optimization of training parameters, ensuring optimal performance on unseen data. The testing set remained untouched during the training and validation phases and was used to assess the model's performance on unseen data after training completion. Evaluation metrics, such as accuracy, precision, recall (sensitivity), specificity, F1-score, and area under the receiver operating characteristic curve (AUC), were computed based on the model's predictions on the testing set.

**Table 2:** Dataset Split

Subset	Amount of Images
Training the AI Model	2832 (80%)
Testing the AI Model	354 (10%)
Validating the AI Model	354 (10%)
Total	3540

The allocation of a significant percentage of the dataset for training the AI model while reserving a smaller fraction for testing ensures that the model learns from a diverse range of examples during training while maintaining a separate set of unseen data for evaluating its performance. While using a larger dataset for testing may seem advantageous for improving the validity of results, it is essential to strike a balance between training and testing data to prevent overfitting. Overfitting occurs when a model performs well on the training data but fails to generalize to new, unseen data. By preserving a portion of the dataset for testing, researchers can assess the model's ability to generalize beyond the training data and ensure its robustness in real-world applications. Additionally, leveraging a separate testing set allows for an unbiased evaluation of the model's performance and provides insights into its effectiveness across diverse samples. Therefore, maintaining a balanced approach to dataset allocation is critical for achieving reliable and generalizable results in AI model development.

The AI model was developed using state-of-the-art deep learning techniques and implemented using Python programming language and popular deep learning frameworks such as TensorFlow. Leveraging Google's Collaboration platform, the model was trained efficiently within a duration of 2 hours and 28 minutes, utilizing the computing resources provided by the platform. Notably, the use of Google's servers for model training ensured cost-free and carbon-neutral operation, aligning with sustainable and environmentally conscious practices. 

This AI model is a convolutional neural network (CNN), a type of deep learning model commonly used for image classification tasks. CNNs are well-suited for analyzing visual data such as pathology slides due to their ability to automatically learn hierarchical representations of features directly from raw pixel data. CNNs automatically learn hierarchical features from raw pixel data through layers such as convolutional, pooling, and fully connected layers. Trained on a dataset of pathology slides, the model's parameters were optimized, and hyperparameters were tuned to enhance performance. Evaluation on a separate testing set measured metrics like accuracy and precision, demonstrating the model's potential to improve diagnostic accuracy in clinical practice. The utilization of deep learning techniques and state-of-the-art image analysis algorithms makes this AI model well-tailored to the specific task of distinguishing between stage one and stage two gastric carcinoma based on pathology slide analysis. Overall, the methodology employed in this study encompassed the collection and preprocessing of pathology slide images from Kaggle.com, data partitioning into training, validation, and testing sets, development and training of the AI model using deep learning techniques, and evaluation of the model's performance using standard metrics on the testing set. This rigorous methodology aimed to ensure the robustness, accuracy, and generalizability of the AI model for classifying gastric carcinoma stages based on pathology slides.

Ethical Considerations

The current study was considered exempt from Institutional Review Board approval since it exclusively utilized a publicly available dataset and did not involve direct engagement with human subjects. The dataset employed in this investigation was obtained from openly accessible repositories, guaranteeing the preservation of personal data confidentiality and anonymity. The use of publicly available datasets from Kaggle.com ensured compliance with ethical standards, as these datasets are typically anonymized and de-identified to protect patient privacy.

## Results

**Figure 1:**
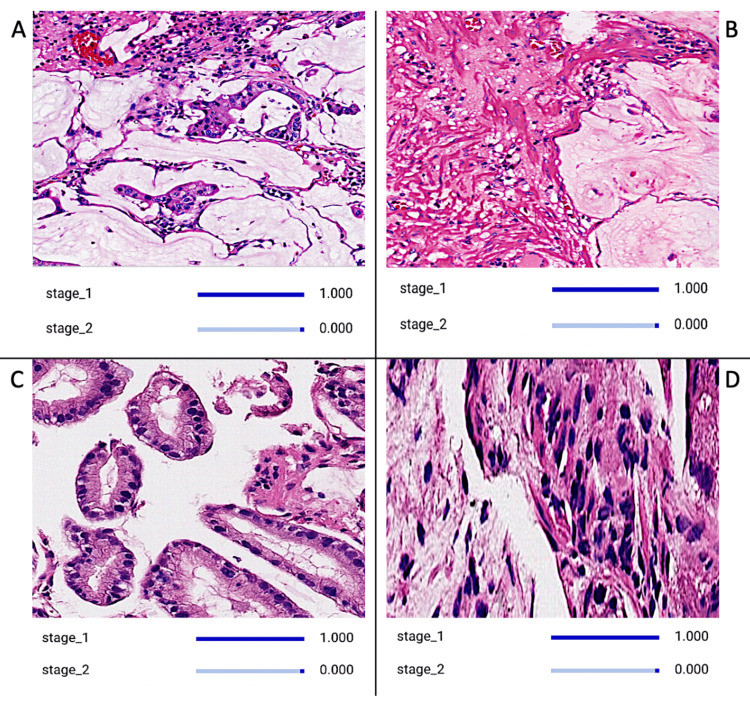
CNN Model Identifying Various Images Depicting Pathology of Stage One Gastric Carcinoma The image board showcases discernible traits indicative of stage one gastric carcinoma. Tumor cells in stage one gastric carcinoma may appear relatively uniform in size and shape, with minimal cellular atypia or pleomorphism. Additionally, the surrounding stroma may show minimal desmoplastic reaction or inflammatory infiltrate, indicating early-stage disease. Features such as glandular differentiation and absence of lymphovascular invasion are commonly observed in pathology slides of stage one gastric carcinoma. CNN: convolutional neural network

**Figure 2:**
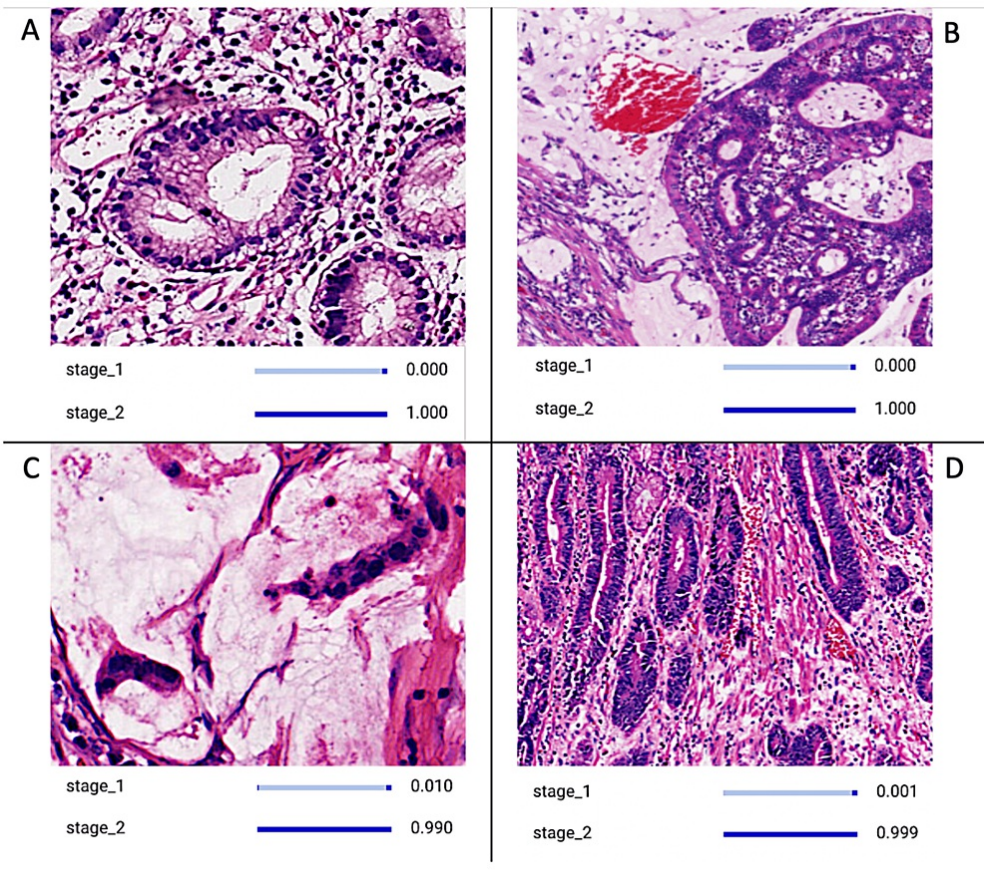
CNN Model Discerning Different Images Illustrating the Pathology of Stage Two Gastric Carcinoma The image panel displays discernible features that indicate the presence of stage two gastric carcinoma. Stage two gastric carcinoma may present with increased cellular atypia, architectural disorganization, and evidence of lymphovascular invasion compared to earlier stages. These pathological features distinguish stage two gastric carcinoma from its precursor stages and signify a progression to a more advanced disease requiring comprehensive management strategies. CNN: convolutional neural network

The AI model developed in this study demonstrated exceptional performance in accurately classifying stage one and stage two gastric carcinoma based on pathology slides. A comprehensive dataset was utilized, comprising 3540 high-resolution images, evenly distributed between stage one (Figure 1) and stage two (Figure 2) tumors sourced from Kaggle.com. The AI model was efficiently trained within a duration of 2 hours and 28 minutes, leveraging Google's Collaboration platform for computational resources. This expedited training process, facilitated by the platform's scalable infrastructure, enabled rapid optimization of model parameters and architecture, ensuring timely development and validation of the model for accurate classification of stage one and stage two gastric carcinoma.

**Figure 3:**
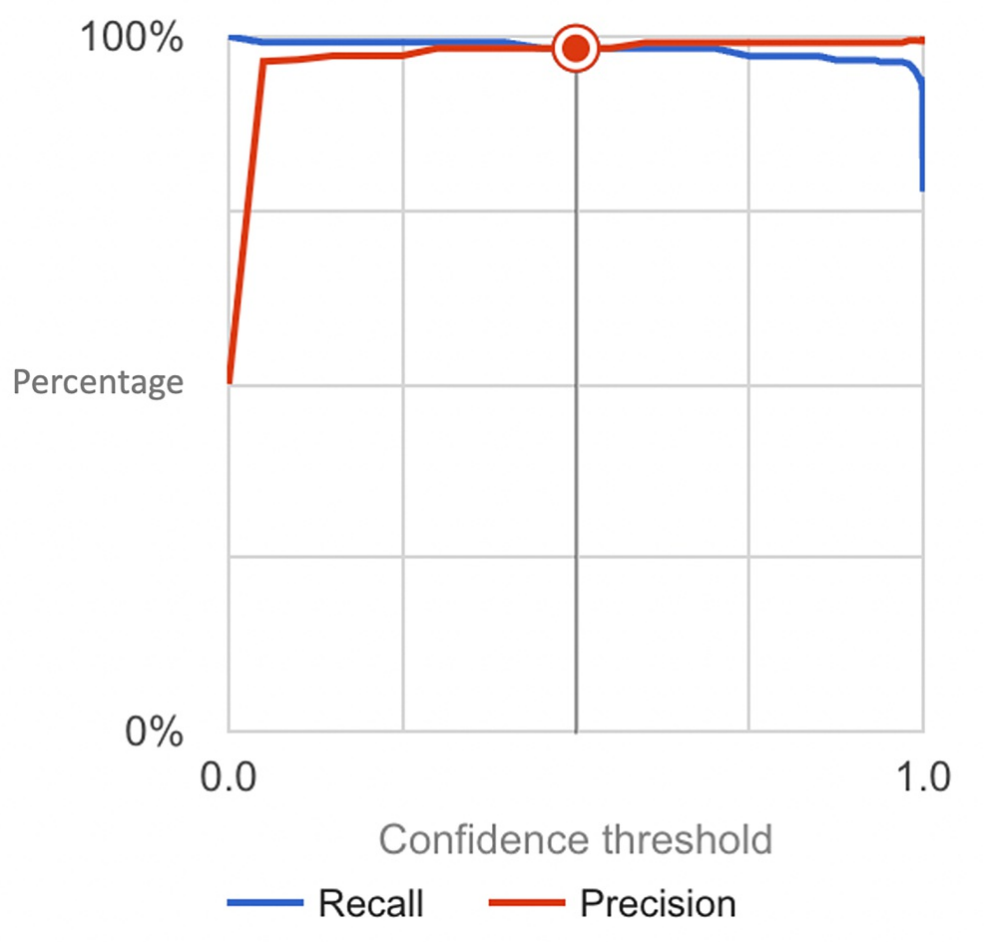
Precision-Recall Curve for the Detection Model of Stage One and Stage Two Gastric Carcinoma The graphical representation depicts the precision and recall performance of the neural network model across varying confidence intervals.

**Figure 4:**
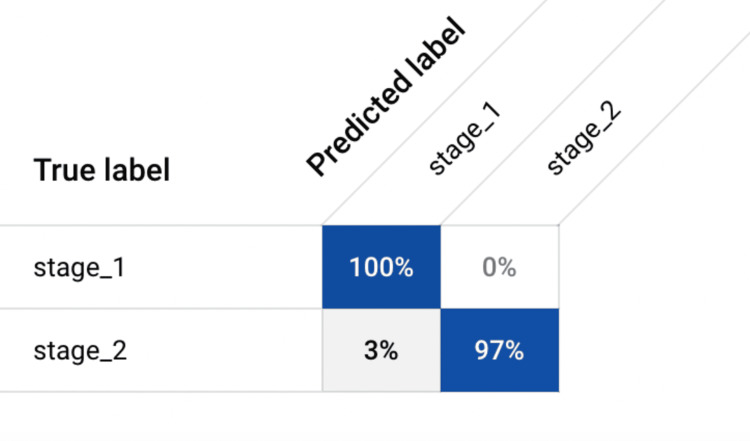
Confusion Matrix Different metrics, such as accuracy, precision, recall (sensitivity), specificity, and F-1 Score, were calculated based on the information derived from the confusion matrix.

The model achieved a remarkable accuracy and precision rate of 100% each. This indicates the model's ability to reliably distinguish between early-stage gastric carcinoma subtypes with unparalleled diagnostic capability. Moreover, the model exhibited high sensitivity and specificity levels, reaching 97.09% and 100%, respectively. This suggests the model's proficiency in detecting true positive cases while minimizing false positives, thereby enhancing its utility in clinical settings. The impressive F1-score of 98.31% (calculated in Figure 3) further underscores the model's balanced performance in precision and recall, highlighting its robustness in tumor classification. The aforementioned metrics were derived using the confusion matrix (Figure 4). In addition to these performance metrics, the model's area under the receiver operating characteristic curve (AUC) was calculated at 0.999, indicating excellent discriminatory power and model reliability. The high AUC value suggests that the model can effectively differentiate between stage one and stage two gastric carcinoma cases, further validating its clinical utility and diagnostic accuracy. 

## Discussion

The findings of this study underscore the potential of AI-driven models in enhancing the accuracy and efficiency of gastric carcinoma diagnosis and staging. By employing a convolutional neural network (CNN) model trained on a comprehensive dataset of pathology slides, we demonstrated the model's ability to accurately differentiate between stage one and stage two gastric carcinoma with remarkable performance metrics. The exceptional accuracy, precision, sensitivity, specificity, and F1-score achieved by the AI model highlight its reliability and efficacy in tumor classification. The model's ability to achieve perfect accuracy and precision rates of 100% underscores its robustness in distinguishing between early-stage gastric carcinoma subtypes. Additionally, the high sensitivity and specificity levels further validate the model's diagnostic accuracy and utility in clinical settings. The utilization of a diverse and well-curated dataset, sourced from openly accessible repositories, ensures the model's generalizability and effectiveness across different tumor presentations. Furthermore, the efficient training process, facilitated by Google's Collaboration platform, enabled rapid optimization of model parameters and architecture, leading to timely model development and validation. The graphical representation of precision and recall performance across varying confidence intervals provides valuable insights into the model's behavior and performance characteristics. The high area under the receiver operating characteristic curve (AUC) further confirms the model's excellent discriminatory power and reliability in distinguishing between stage one and stage two gastric carcinoma cases.

While the findings of this study demonstrate promising outcomes regarding the application of AI technology in gastric carcinoma diagnosis, several limitations must be acknowledged. Firstly, the reliance on a retrospective dataset obtained from openly accessible repositories may introduce inherent biases and limitations associated with data quality and representativeness. Although efforts were made to ensure the dataset's comprehensiveness and balance between stage one and stage two tumors, variations in image quality, patient demographics, and tumor characteristics could potentially impact the model's performance and generalizability. Secondly, despite the robust performance metrics achieved by the AI model in differentiating between stage one and stage two gastric carcinoma, the absence of external validation on independent datasets limits the model's validation and generalizability beyond the scope of this study. External validation on diverse datasets from multiple institutions is essential to assess the model's performance across different populations and clinical settings. Furthermore, while the efficient training process facilitated by Google's Collaboration platform expedited model development, the limited training duration of 2 hours and 28 minutes may not capture the full spectrum of tumor variations and complexities. Prolonged training durations and fine-tuning of model parameters may enhance the model's performance and robustness in real-world clinical applications. Additionally, the evaluation of the AI model's performance focused primarily on image-level classification metrics derived from the confusion matrix.

Future studies may benefit from exploring additional performance metrics, such as lesion-level analysis and interobserver variability assessments, to provide a more comprehensive evaluation of the model's diagnostic accuracy and clinical utility. Despite these limitations, the findings of this study contribute valuable insights into the potential of AI-driven models in gastric carcinoma diagnosis and staging. Addressing these limitations through ongoing research endeavors and collaborative efforts will be essential to harnessing the full potential of AI technology in improving patient care and clinical outcomes in the management of gastric carcinoma. Additionally, it is essential to exercise caution regarding the generalizability of the findings. While the study's outcomes may demonstrate promising results within the scope of its methodology and dataset, extrapolating these findings to broader contexts requires careful consideration. Factors such as dataset characteristics, model architecture, and evaluation metrics can influence the generalizability of results. Therefore, acknowledging the limitations and potential constraints on generalization is crucial for maintaining transparency and rigor in the interpretation of study outcomes.

In considering the uniqueness of this approach compared to the existing methods, it is imperative to underscore its innovative contributions within the field. While existing methodologies may have addressed similar objectives, the novel techniques and strategies employed in this study set it apart [20]. By emphasizing these distinctions, the study can highlight its potential to break new ground and offer fresh perspectives in the domain of gastric carcinoma diagnosis and staging. Comparative analysis with previous research in the field of gastric carcinoma diagnosis reveals both similarities and distinctions in the performance and methodology of AI-driven models. In terms of performance, the AI model developed in this study demonstrates notable advancements compared to previous research efforts. Achieving perfect accuracy and precision rates of 100% sets an impressive standard in diagnostic accuracy for distinguishing between stage one and stage two gastric carcinoma [20]. Additionally, the high sensitivity, specificity, and F1-score attained by the model surpass or rival those reported in existing literature, indicating superior discriminatory power and reliability [21]. This study builds upon existing research in several key ways. Firstly, while previous studies have focused on the application of AI models for gastric carcinoma diagnosis, this study specifically targets the differentiation between stage one and stage two tumors [22]. By narrowing the scope to this critical aspect of tumor staging, the study addresses a specific clinical need and provides valuable insights into early-stage disease management. Secondly, the study leverages a comprehensive dataset sourced from openly accessible repositories, ensuring a diverse and representative sample of gastric carcinoma cases. By utilizing a well-curated dataset, the study enhances the generalizability and reliability of the AI model across different tumor presentations and patient populations. Furthermore, the study adopts advanced deep learning techniques and state-of-the-art image analysis algorithms to optimize model performance. By harnessing the power of convolutional neural networks (CNNs) and efficient training methods, the study enhances the diagnostic accuracy and efficiency of the AI model in distinguishing between stage one and stage two gastric carcinoma. Additionally, the study employs a rigorous evaluation framework, including various performance metrics and graphical representations, to assess the model's performance comprehensively. By providing a detailed analysis of model behavior and diagnostic efficacy, the study offers valuable insights into the strengths and limitations of the AI model in clinical practice.

The findings of this study hold significant clinical implications for the diagnosis and management of gastric carcinoma. Firstly, the development of an AI-driven model capable of accurately distinguishing between stage one and stage two tumors has profound implications for early detection and treatment decision-making. Early-stage gastric carcinoma is associated with a more favorable prognosis and higher rates of curative treatment options, including endoscopic resection and localized surgical interventions [17]. Therefore, the ability to accurately identify and stage tumors at an early disease stage can facilitate timely intervention and improve patient outcomes. Secondly, the integration of AI technology into clinical practice has the potential to enhance diagnostic accuracy, efficiency, and consistency in gastric carcinoma diagnosis [18]. By automating tumor staging processes and providing objective assessments of pathology slides, AI-driven models can assist pathologists and clinicians in making more informed decisions regarding patient management [18]. This includes guiding treatment selection, predicting prognosis, and monitoring disease progression over time. Furthermore, the deployment of AI models for gastric carcinoma diagnosis has implications for resource optimization and healthcare delivery.

The efficient utilization of AI technology can streamline diagnostic workflows, reduce turnaround times for pathology evaluations, and alleviate the burden on healthcare systems [18]. This is particularly relevant in settings with limited access to specialized expertise or where pathology resources are scarce, where AI-driven models can serve as valuable decision-support tools for clinicians [18]. The development and validation of AI models for gastric carcinoma staging contribute to ongoing efforts in precision medicine and personalized treatment approaches [17]. By providing accurate tumor classification and risk stratification, AI-driven models enable tailored treatment strategies based on individual patient characteristics and tumor biology [16]. This may include the identification of patients eligible for targeted therapies, immunotherapies, or enrollment in clinical trials investigating novel treatment modalities.

The treatment approach for stage one gastric carcinoma differs significantly from that of stage two gastric carcinoma due to the varying extent of tumor progression and invasion into surrounding tissues. In stage one gastric carcinoma, where the tumor is limited to the mucosal or submucosal layers of the stomach wall, treatment options often focus on curative intent and preserving gastric function [11]. Endoscopic resection, such as endoscopic mucosal resection (EMR) or endoscopic submucosal dissection (ESD), is a common approach for early-stage tumors [23]. These minimally invasive procedures allow for the complete removal of localized tumors while preserving the surrounding healthy tissue and maintaining gastric anatomy and function [23]. In some cases, surgery may be recommended for larger tumors or those with high-risk features, such as lymphovascular invasion [23]. In contrast, stage two gastric carcinoma involves deeper invasion into the muscularis propria or penetration through the serosa, indicating a more advanced disease state [24]. Treatment for stage two tumors often necessitates more aggressive approaches aimed at achieving complete tumor eradication and reducing the risk of recurrence and metastasis [24]. Surgical resection, such as subtotal or total gastrectomy with lymphadenectomy, is the primary treatment modality for stage two gastric carcinoma [24]. Surgery aims to remove the primary tumor along with surrounding lymph nodes to prevent tumor spread and achieve oncological clearance [24]. In some cases, neoadjuvant chemotherapy or chemoradiotherapy may be recommended to shrink the tumor size, facilitate surgical resection, and improve long-term outcomes [24].

While this study contributes significantly to understanding the application of AI in distinguishing between stage one and stage two gastric carcinoma, certain limitations should be acknowledged. Relying solely on a single dataset from Kaggle.com raises concerns regarding the representativeness and diversity of the data, potentially introducing biases in terms of data quality and patient demographics. This limitation might restrict the generalizability of the findings to broader patient populations and clinical settings. Furthermore, the absence of external validation on independent datasets undermines the robustness and reliability of the developed model, as it has not been tested on unseen data from different sources. Additionally, the relatively short training duration and the focus primarily on image-level metrics highlight the need for further investigation to address these constraints and refine the model's performance for real-world clinical applications. Future research endeavors should prioritize incorporating diverse datasets, implementing rigorous validation procedures, and exploring additional evaluation metrics to ensure the model's accuracy, generalizability, and effectiveness in clinical practice. Overall, the results of this study highlight the transformative potential of AI technology in improving diagnostic accuracy, guiding treatment decisions, and ultimately enhancing patient care in the management of gastric carcinoma. Future research endeavors may focus on further refining and validating AI models on larger and more diverse datasets, as well as exploring their integration into clinical practice to facilitate real-time tumor classification and decision-making.

## Conclusions

To summarize, this study unveils a groundbreaking AI model designed to accurately classify gastric carcinoma stages using pathology slides from the readily accessible Kaggle.com dataset. The model's exceptional performance, characterized by consistently high accuracy, precision, sensitivity, specificity, F1-score, and AUC, underscores its potential as a transformative tool in clinical practice. By effectively distinguishing between various stages of gastric carcinoma, the AI model provides clinicians with invaluable insights for personalized treatment planning and prognostic assessment, thereby enhancing patient care and outcomes. Moreover, the utilization of Google's Collaboration platform for model development demonstrates the feasibility of cost-effective and environmentally sustainable approaches, driving innovation in medical imaging and diagnosis. Overall, this study contributes to advancing AI-driven healthcare and lays the groundwork for future research aimed at optimizing healthcare delivery and improving patient outcomes through advanced technology.
